# Rest in Rheumatism

**Published:** 1900-04-07

**Authors:** 


### REST IN RHEUMATISM.

As at the meeting of the Clinical Society which we recently commented upon, so during a discussion on the treatment of rheumatism which occupied the last two meetings of the Chelsea Clinical Society, much emphasis was laid upon the enforcement of complete rest. Sir R. Douglas Powell said he thought that the profession hardly yet grasped the exceeding importance of absolute and prolonged rest in the treatment of rheumatic fever, especially, of course, in cases in which the heart was involved; and he thought that the statistics of the salicylate treatment had been largely affected by the failure to ensure rest after relief had been obtained by that drug. A practitioner would, he said, give salicylates to a patient, with the result that in two or three days the pain was gone, and the patient did not see why he should not get up, and in such cases much determination was required to keep him at rest. Salicylate treatment, he said, relieves the symptoms of the disease and subdues many of its important features, but it does not cure the rheumatic illness, nor does it shorten its duration; and if because the pain is relieved the patient begins to move about while he still has the disease in a latent form he may have a relapse, and in all probability a relapse with cardiac complications. The point in the treatment of rheumatism is that so long as the tongue is coated, the urine is loaded, and there is the slightest trace of pyrexia, and also for a period of a good many days afterwards, people must be kept rigidly in bed, in warm clothing, even in the complete absence of any cardiac affection. In regard to this question of pyrexia also there is a point that is of importance. One often hears that a patient's temperature only runs up to about 99 deg. Fahr., or a little below, and one is told that he is not feverish. But 99 deg. Fahr. in the mouth or the axilla is a morbid degree of temperature, and taken in association with recent rheumatism or a slight endocarditis is an absolute sign of the necessity of preserving complete rest. Recurrence is more to be feared than the primary attack, for the two most grave cardiac affections of rheumatic origin—namely, mitral stenosis and aortic regurgitation—are scarcely ever met with in primary rheumatism, but almost always in cases which have left the hospital, and have returned again some months later. These two lesions are the consequences of subsequent slow-deforming valvulitis from the strain put upon the valves before they have competely recovered from the primary affection.

